# Functionalization of screw implants with superelastic structured Nitinol anchoring elements

**DOI:** 10.1186/s12938-021-00975-4

**Published:** 2022-01-11

**Authors:** Isabell Hamann, Stefan Schleifenbaum, Christian Rotsch, Welf-Guntram Drossel, Christoph-Eckhard Heyde, Mario Leimert

**Affiliations:** 1 Fraunhofer Institute for Machine Tools and Forming Technology IWU, 01187 Dresden, Germany; 2 Asklepios Orthopädische Klinik Hohwald, 01844 Neustadt in Sachsen, Germany; 3 Department of Orthopedic Surgery, Traumatology and Plastic Surgery, Leipzig University, 04103 Leipzig, Germany; 4 ZESBO—Zentrum zur Erforschung der Stuetz- und Bewegungsorgane, Leipzig University, 04103 Leipzig, Germany; 5 Chemnitz University of Technology, 09107 Chemnitz, Germany; 6 Sächsische Schweiz Kliniken GmbH, 01855 Sebnitz, Germany

**Keywords:** Screw implants, Superelasticity, Additive manufacturing, Biomechanic, Nitinol

## Abstract

**Background:**

Demographic change is leading to an increase in the number of osteoporotic patients, so a rethink is required in implantology in order to be able to guarantee adequate anchoring stability in the bone. The functional modification of conventional standard screw implants using superelastic, structured Ti6Al4V anchoring elements promises great potential for increasing anchoring stability.

**Methods:**

For this purpose, conventional screw implants were mechanically machined and extended so that structured-superelastic-positionable-Ti6Al4V anchoring elements could be used. The novel implants were investigated with three tests. The setup of the anchoring elements was investigated in CT studies in an artificial bone. In a subsequent simplified handling test, the handling of the functional samples was evaluated under surgical conditions. The anchorage stability compared to standard screw implants was investigated in a final pullout test according to ASTM F543—the international for the standard specification and test methods for metallic medical bone screws.

**Results:**

The functionalization of conventional screw implants with structured superelastic Ti6Al4V anchoring elements is technically realizable. It was demonstrated that the anchoring elements can be set up in the artificial bone without any problems. The anchorage mechanism is easy to handle under operating conditions. The first simplified handling test showed that at the current point of the investigations, the anchoring elements have no negative influence on the surgical procedure (especially under the focus of screw implantation). Compared to conventional standard screws, more mechanical work is required to remove the functional patterns completely from the bone.

**Conclusion:**

In summary, it was shown that conventional standard screw implants can be functionalized with Ti6Al4V-structured NiTi anchoring elements and the new type of screws are suitable for orthopedic and neurosurgical use. A first biomechanical test showed that the anchoring stability could be increased by the anchoring elements.

## Introduction/background

Demographic change is leading to an increase in patients with spinal disorders and therefore to an older population with greater and degenerative comorbidities (e.g., osteoporosis). Thus, the number of spine procedures has increased. This circumstance makes it difficult for patients to enable optimal and stable implant fixation in the bone [1–3]. Therefore, the focus of implant research is currently on the further development and adaptation of screw implants to increase anchorage stability in osteoporotic bone without the use of bone cement. An innovative approach is the use of erectable anchoring elements that increase the screw cross-section and thus minimize loosening phenomena [4]. The use of thermally activated and superelastic Nitinol (NiTi) for anchoring elements is a promising approach. Its special properties—one-way, two-way effect or deformation by up to 8% from the initial position [5]—enable the functionalization of conventional standard implants to the development of new implants and instruments. In particular, the high deformation of the superelastic Nitinol allows a large revisable adjustment range of functional anchorage elements [5]. Especially because of its good biocompatibility, it is suitable for use in the human body [6, 7].

Initial approaches showed that the use of thermally activated NiTi as an anchoring element has great potential for increasing anchorage stability [4].

A deficit of thermal NiTi anchoring elements is a complex and expensive temperature management (cooling during assembly, storage and implantation) [4]. Furthermore, although its smooth surface of the NiTi anchoring elements already causes an increase in anchorage stability, a textured (enlarged) surface could increase anchorage stability even further.

To address this, anchoring elements were manufactured with superelastic Nitinol and structured with medical titanium grade alloy Ti6Al4V elements. The structuring of the NiTi sheets is produced by using the laser powder bed fusion (LPBF) technology manufacturing process, which is already established in medical technology [8, 9]. In this process, Ti6Al4V powder is fused onto the NiTi anchoring element via the laser beam process. The manufacturing process is already state of the art and is mainly used for patient-individualized implant fabrications or for implant functionalizations [8, 10, 11]. Ti6Al4V is a conventional implant material and is also biocompatible in its powder form and suitable for use in the human body [8, 10]. In order to realize the largest possible, but also safest surface increase and thus improved anchoring in the bone, pyramid-shaped structures were fabricated on the NiTi sheet. Various geometries for the structures were examined in mathematical and simulative investigations. In the investigations of the surface enlargement (largest possible interlocking), the connection area to the NiTi element (largest possible area between NiTi element and Ti6Al4V structure for optimal hold) and the Von Mises stress in the load case, the pyramid-shaped structures achieved the optimal results.

The use of superelastic instead of thermally activated NiTi eliminates the need for complex temperature management. In addition, the revision process (removal of the implant with as little damage to bone tissue as possible) is improved and the level of change in geometry of the anchoring elements is increased due to the superelasticity.

The aim of the present study was to functionalize standard screw implants in order to increase anchorage stability in bone and revision capability by using superelastic, structured anchoring elements and to investigate them with regard to their handling under surgical conditions in artificial and human bone.

## Results

### NiTi-screw

**Fig. 1:**
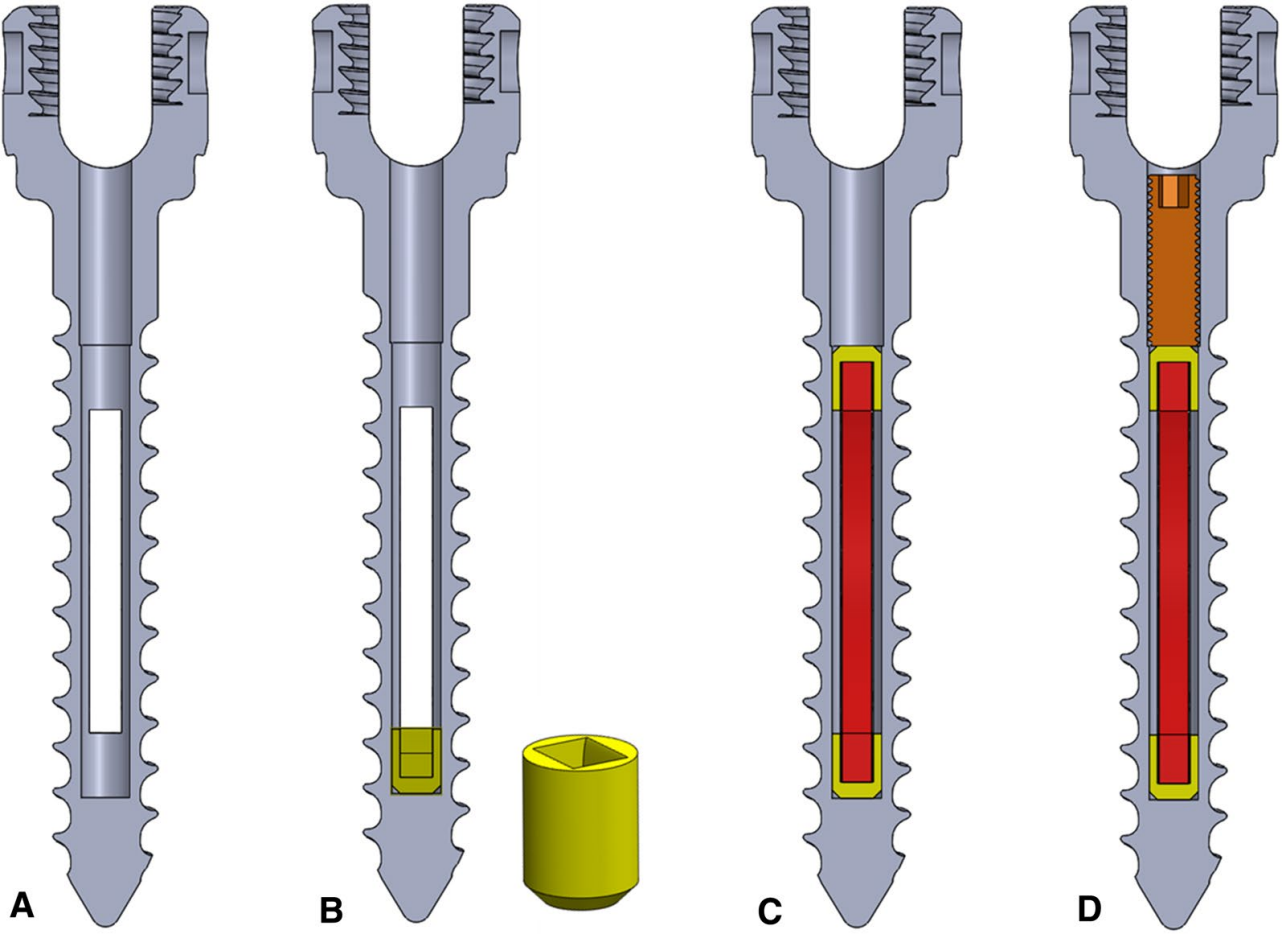
Structure of the NiTi screw. **A** screw base body with hole; **B** additively manufactured guide sleeve (yellow); **C** structured NiTi anchor element (red) with guide sleeves (yellow); *D* threaded cone (orange)

For the integration of NiTi anchoring elements, conventional standard monoaxial pedicle screws (45  ×  6.5 mm) were drilled with a 40-mm-long hole (*ø* 3 mm) and two opposing identical pockets were eroded from the base body. In addition, a M3.5 thread with a depth of 5 mm was countersunk into the hole (Fig. 1A).

The anchoring elements were made of 0.3-mm-thick, 1.8-mm-wide and 20-mm-long superelastic Nitinol from Ingpuls GmbH, Germany, which were structured with 0.5  ×  0.5 × 1 mm Ti6Al4V-pyramids. For the assembly and guidance of the NiTi anchoring elements (red) inside the base body, titanium guide sleeves (yellow) were additively manufactured (Concept Laser M2 Cusing, Germany); see Fig. 1B and C. A standard threaded cone (orange) is used as the final component, which realizes the positioning of the sheets and at the same time acts as an anti-twisting device (Fig. 1D).

### Proof of function

#### Test 1: Functional proof of the installation mechanism

**Fig. 2:**
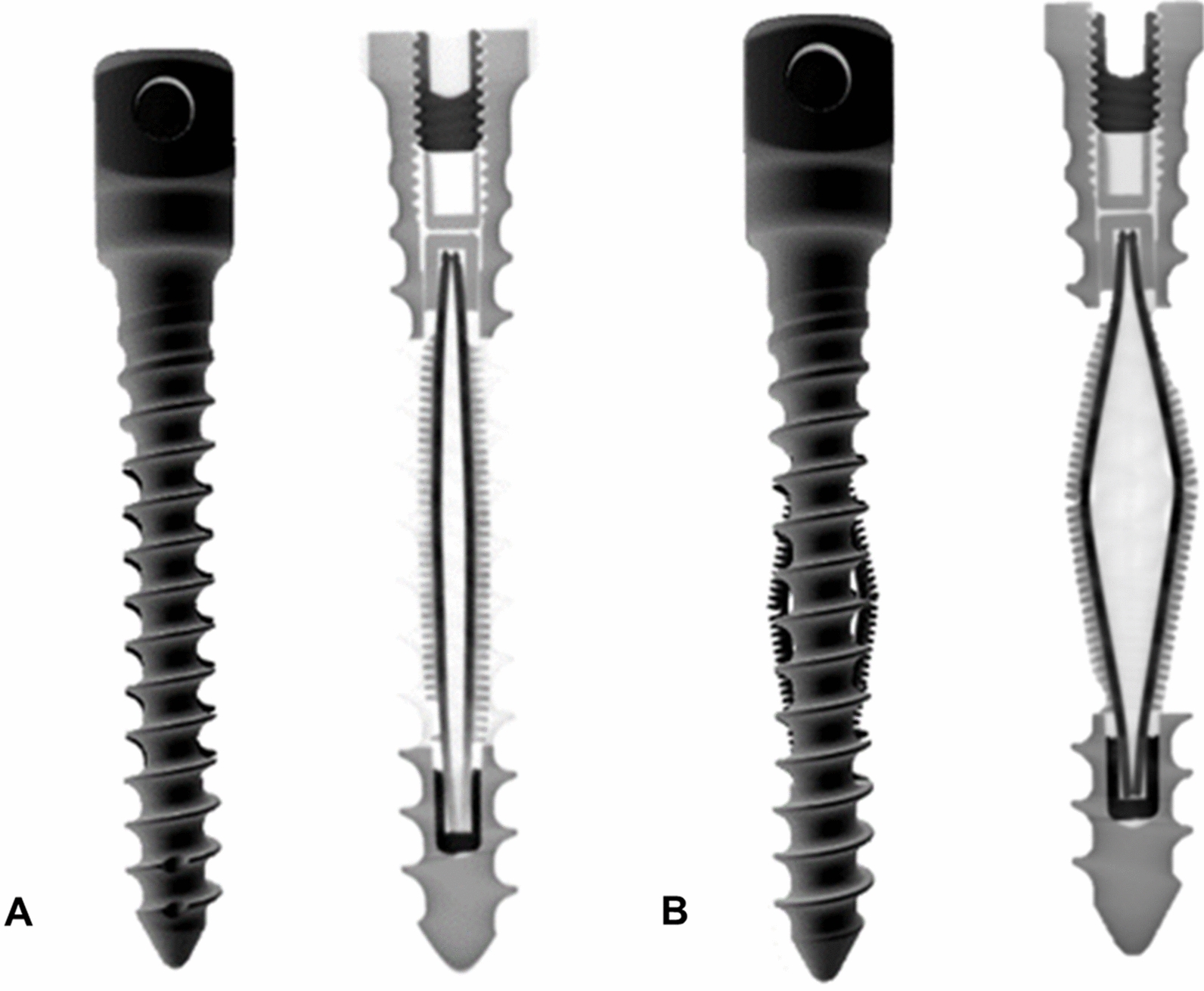
CT-images of the NiTi-screw. **A** After implantation; **B** after setup the anchoring elements

The setup mechanisms of the NiTi anchoring elements in artificial bone were successfully demonstrated in a CT (Fig. 2).

#### Test 2: Handling test

The evaluation of the questionnaire showed that there were no haptic differences in the handling and feel of the NiTi screws during implantation compared to conventional, unmachined screw implants. The eroded pockets do not affect the stability of the screw in the bone. Despite the absence of a thread, no differences in the haptic and objective evaluation of the anchorage stability compared to a conventional screw were observed after implantation (before the setup) of the functionalized screw. The haptic comparison of the primary stability, after the setup of the NiTi screws, compared to the cement augmentation was rated by one of the two neurosurgeons with 7 out of 10 possible points (10 = high primary stability) and a noticeable improvement was observed. Neurosurgeon two abstained from comment.

The additional effort to set up the anchoring elements due to the rotation mechanism can be negligible.

The rotation mechanism for positioning the anchoring elements was rated as intuitive, so that very good haptic feedback was felt when the anchoring elements were applied to the bone. The torque was also rated as just right. It was not too low and not too high to allow optimal adjustment that allows the user to feel when the anchoring element is applied to the bone and compresses without having to apply too much force. It was well adjusted especially for osteoporotic bone tissue.

**Fig. 3:**
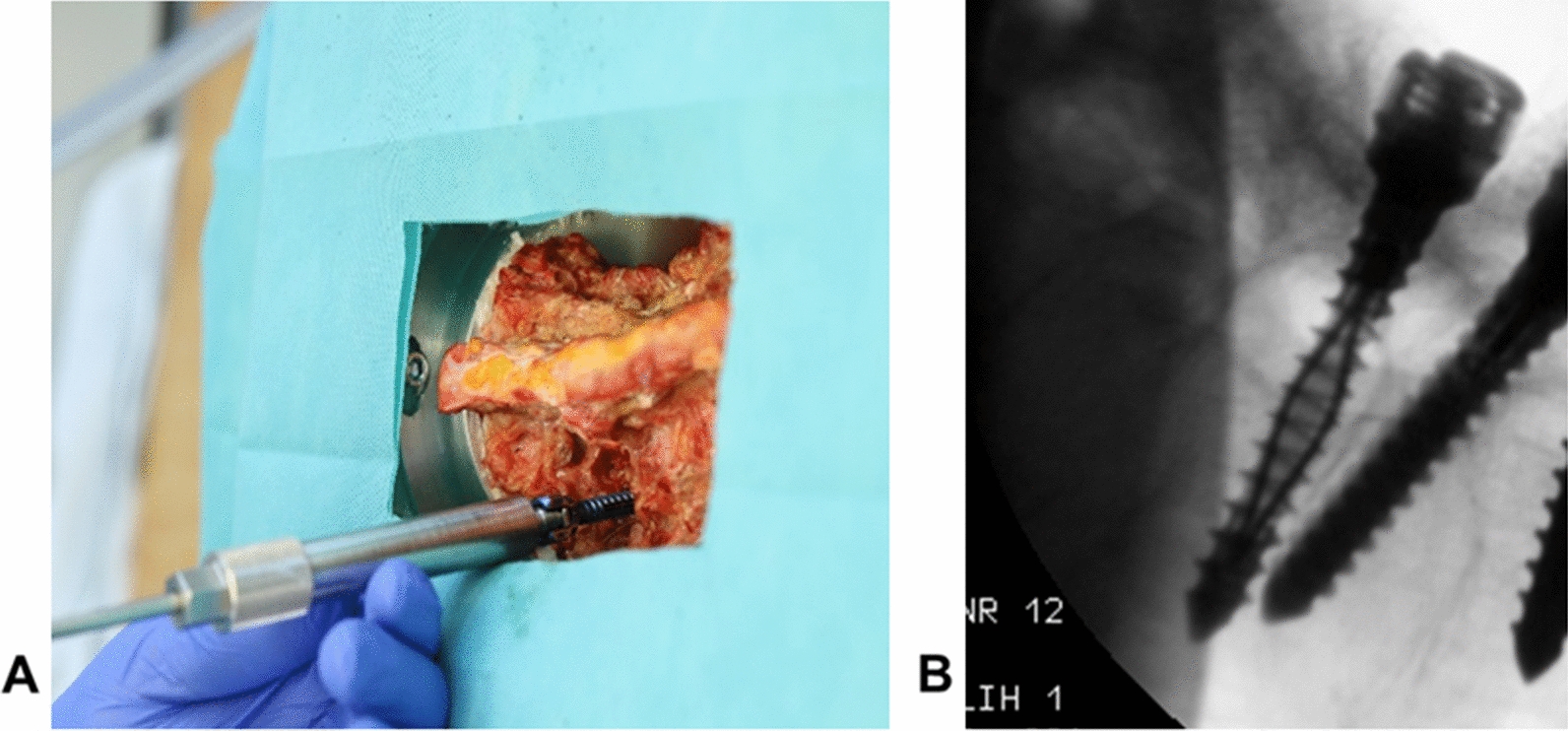
Handling test. **A** Implantation of the NiTi screw; **B** CT image of the implanted NiTi screw

To check the position of the screws a C-arm (Ziehm Imaging, Nuremberg, Germany) was used. Clear imaging without artifacts of the NiTi screws as a whole as well as of the individual anchoring elements was demonstrated with this (Fig. 3).

#### Test 3: Functional proof of the anchoring mechanism

Preliminary results of the pullout test in artificial bone showed a tendency for anchorage stability increase of the NiTi screw compared to standard screws.

**Fig. 4:**
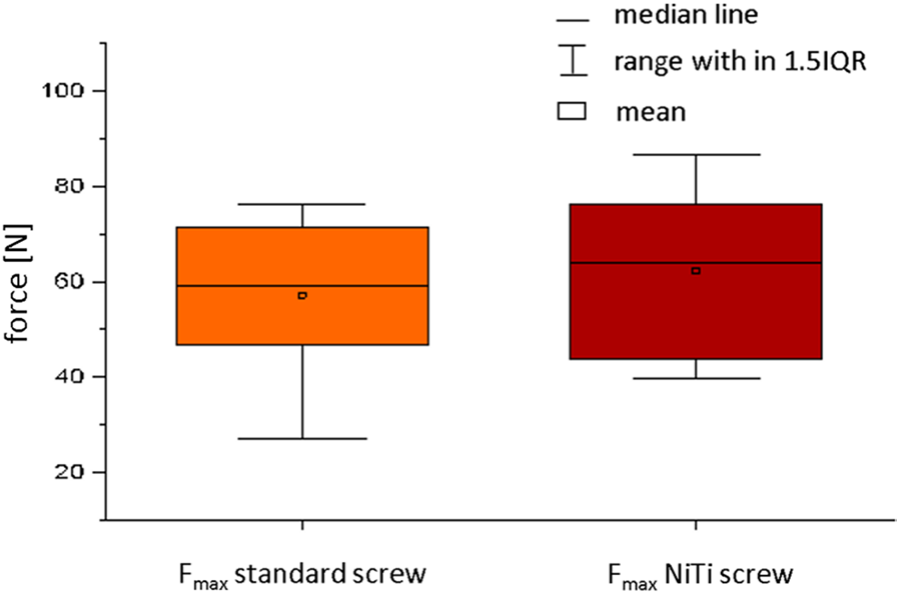
Pullout force of the standard screw (orange) and NiTi screw (red)

The pullout force of the standard screws was 57.22 N  ±  17.13 N and the pullout force of the NiTi screws was 62.36 N  ±  18.55 N (Fig. 4)

**Fig. 5:**
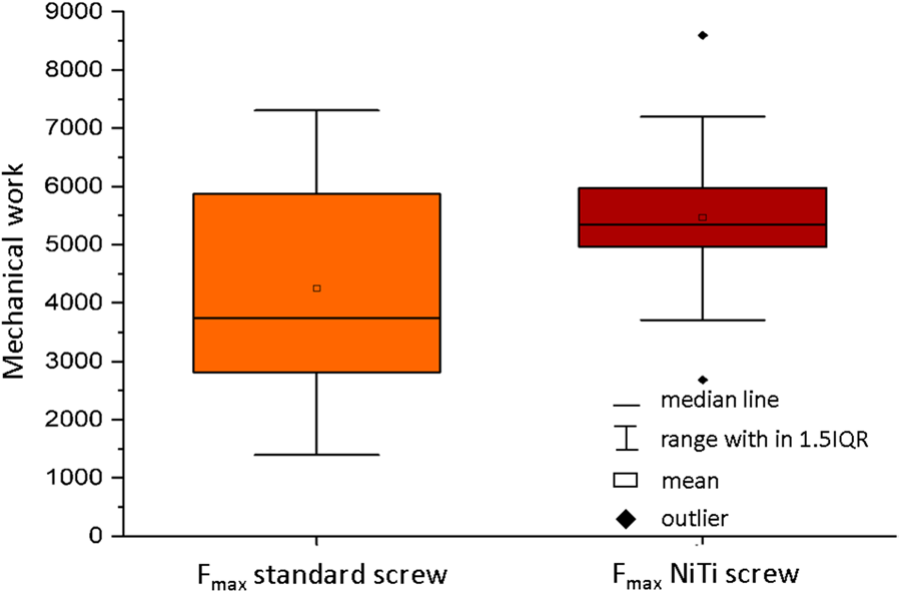
Mechanical work of the standard screw (orange) and NiTi screw (red)

The mechanical work (energy that must be applied to break out the screw) of the standard screws was 4241.541 Nm  ±  42.549 Nm and that of the newly developed screws was 5464.796 Nm  ±  435.508 Nm (Fig. 5).

## Discussion

In our study, it was shown that a functionalization of conventional screw implants using Ti6Al4V-structured NiTi anchoring elements is implementable. Furthermore, it could be demonstrated that the NiTi screws are suitable for pre-clinical tests and are not inferior to standard screw implants.

In addition to the production-related investigation of the integration of the NiTi anchoring elements in standard screws, it was particularly important to evaluate whether the functionalization can be implemented for larger batch sizes. This is an important factor in satisfying the increasing demand for alternative screw implants for patients with osteoporosis due to demographic change [12, 13] and for reducing their manufacturing costs [14].

The screw base body could be machined using conventional standardized manufacturing technologies such as drilling and EDM (electrical discharge machining), so that no further special tools were required. These process steps are currently already applicable for serial production [15]. It would also be conceivable to use a milling machine for the deep hole drilling and the production of the pockets for the anchoring elements. This could combine the two individual steps and thus reduce manufacturing time and effort [15–17].

The sleeves, which are additively manufactured for assembly, are of significant advantage for guiding the NiTi elements, as they align the elements and fix them against twisting during implementation.

The sleeves have a recess that can accommodate two NiTi anchoring elements and hold them hold them parallelly aligned A clearance fit allows them to be inserted into the screw and rotate, so that the anchoring elements shift into the correct position with regard to the pockets.

Serial production is possible because of the small components and the associated large batch size on the build plate [18–20]. Moreover, reworking such as deburring and polishing the sleeves is not necessary. Additive manufacturing is an additional advantage here because medically biocompatible Ti6Al4V is used, which is approved for use in medical technology [8].

In addition, the geometry of the sleeves can be made and adapted to the given implants without additional tools and complex design planning [20, 21]. Additive manufacturing is therefore already considered forward-looking in medical technology [21].

The anchoring elements made of superelastic NiTi can be “stretched 10 times more than conventional spring steels without permanent, i.e., plastic, deformation” [22] and thus enable a large setup travel (maximum bending of the elements) and improved revision (resetting of the elements after loosening the rotation mechanism without plastic deformation). Due to these properties, the NiTi elements can be stretched up to 8% [5, 23], which results in a maximum setup travel (s) per element of *s* = 17.7 mm (calculation according to DIN ISO 178 [24]).

However, this is limited by the applied Ti6Al4V structures, which have a lower elasticity and can flake-off if the deflection is too large [25]. Therefore, a limitation of the anchoring mechanism is necessary to prevent excessive deflection.

Furthermore, the superelastic properties improve revision surgery. It allows for a more bone-sparing revision therapy (compared to bone cement augmentation), because by releasing the anchoring mechanism, the anchoring elements return to the screw without plastic deformation and occupy their original position at implantation (inside the screw). This unsets the cross-sectional enlargement and allows a more bone-sparing revision. In addition, there is no need for complex temperature management compared to thermally activated NiTi elements.

The structuring of the NiTi anchoring elements with Ti6Al4V by the LSM process can be implemented satisfactorily and the fabrication over large areas is possible [18]. In a previous study by our group, it was already proven that the connection of two established implant materials (Nitinol and Ti6Al4V) by means of the LSM process is biomechanically suitable [26]. Because of the high proportion of titanium in Nitinol, a good connection could be achieved. Another of our studies has already shown that the structuring technology of the anchoring elements is suitable for implantology due to its good biocompatibility [27].

The structuring of the anchoring elements was chosen so that the greatest possible interlocking can take place. The larger the surface of the structure, the greater the interlocking effect [28, 29]. In terms of its optimum mechanical force dissipation, with an acting surface force, the pyramid structure was therefore chosen.

Our investigation showed that the NiTi anchoring elements could be inserted and set up in the artificial bone. This served to prove that even with surrounding harder tissue, the anchoring elements could be placed without complications and that no tilting occurred during placement.

In a further step, it was shown that the anchoring elements could also be placed in a human specimen without any problems. Surrounding bone and tissue structures did not cause disruption of the mechanism. One advantage of the mechanical setup was that it was haptically detected when the anchoring element was applied to the bone (increase in torque). The surgeon thus has direct feedback on the anchorage status, which was considered positive.

At the same time, the experiments showed that the NiTi elements did not affect the imaging. No radiation overlaps occurred, they were easily controllable in lateral X-rays and the setup mechanisms could be evaluated without restrictions. This is particularly important for neurosurgical and orthopedic use, as the screw implants and their anchoring elements must not over-radiate surrounding tissue structures because of proximity to the spinal canal [30–32].

Furthermore, the simplified handling test and the evaluation of the functional samples by the surgeons showed that the NiTi screws—despite the missing thread due to the eroded pockets—could be compared with standard screws during implantation (compressed spongiosa in the pedicle) and that there was no haptic difference. This is particularly important for users, as they can continue to rely on their typical haptic feel and experience when screwing in screw implants.

The haptic feel is also important for the correct torque when setting up the anchoring elements and it could be optimally reproduced with the present mechanism. In this way, the surgeon can already detect an increase in stability during setup. For an initial assessment at this stage of development and the associated small number of available functional samples, only a small number of test implantations could be carried out. Further investigations in human and animal bone structures are planned in order to be able to make a more statistical statement.

The screw extraction test according to ASTM F543 [33] showed that despite the lack of significance of the increase in extraction force, the mechanical work performed to extract the implant increased. A tendency could be demonstrated that the functionalized screws require more force and energy to extract the implant from the artificial bone, to remove the screw and to loosen it compared to the standard screws. The use of artificial bone material makes it difficult to make a clear statement. The material has a foam-like structure, so that not every block is clearly reproducible with regard to its mechanical properties and thus mechanical and material deviations can occur. Because of the additionally increased surface due to the structuring of the anchoring elements, these deviations can be amplified and outliers, as seen in Fig. 5, can occur. In order to relativize the deviations, more random samples have to be taken. In addition, an instrument must be developed that enables a reproducible setup (adjustment of the angle of rotation) of the setup elements. This was carried out in this study by the surgeon in each case. For initial preliminary examinations regarding the increase of anchorage stability (proof of tendency) and proof of function (placement of the anchorage elements), the artificial bone material is sufficiently suitable. In further studies on artificial bone and human preparations, a higher number of samples have to be used. In order to test the functional principle (setup in artificial bone and human specimens) and, if necessary, to record constructive adjustments regarding screw and anchoring element design, only a small number of screw functional samples were available for testing at this stage of the study. In addition, further investigations, such as toggling tests in human specimens, are currently being carried out in order to optimally simulate and evaluate the anchoring stability under biomechanical movements.

## Conclusion

In summary, conventional monoaxial standard screws can be functionalized with Ti6Al4V-structured NiTi anchoring elements. The fabrication of the screw base body and integration of the NiTi anchoring elements is suitable for series production and the handling does not differ from standard screws. A first biomechanical test showed that the anchoring stability can be increased by the anchoring elements.

## Methods

The aim of the present study was to functionalize standard screw implants to increase anchorage stability in bone and revision capability by using superelastic, structured anchorage elements.

### NiTi-screw

To functionalize the screw implants, standard monoaxial pedicle screws were refined, mechanically processed and anchoring elements were used along the principles of the dowel and based on our own preliminary work [4]. Superelastic NiTi was used for the base of the anchoring elements and structured with Ti6Al4V to increase the surface area and thus the interlocking effect. The structuring of the NiTi elements was implemented using the manufacturing process of laser beam melting with Ti6Al4V. Based on our own preliminary investigations regarding the manufacturing strategy [26], the titanium structures were manufactured with a laser power of 130 W; scanning speed: 1000 mm/s and respective average metallurgical melting depth: 33.3 µm.

The aim of the study was to investigate the increase of anchorage stability of screw implants by using superelastic anchorage elements with pyramid structure made of Ti6Al4V with regard to their manufacturing, handling and biomechanical pullout test.

### Proof of function

#### Test 1: Functional proof of the installation mechanism

The setup mechanisms of the structured NiTi anchoring elements were tested in an artificial bone. For this purpose, the screw functional samples were implanted in a pre-drilled artificial bone cube with unset anchoring elements. Subsequently, the anchoring elements were set up so that a diameter increase of 2 mm results in the non-implanted state. For the evaluation and verification of the setup of the anchoring elements, the artificial bone cuboid with setup anchoring structure was analyzed in a µCT (vItomeIx s, GE Sensing & Inspection, Germany).

#### Test 2: Handling test

**Fig. 6:**
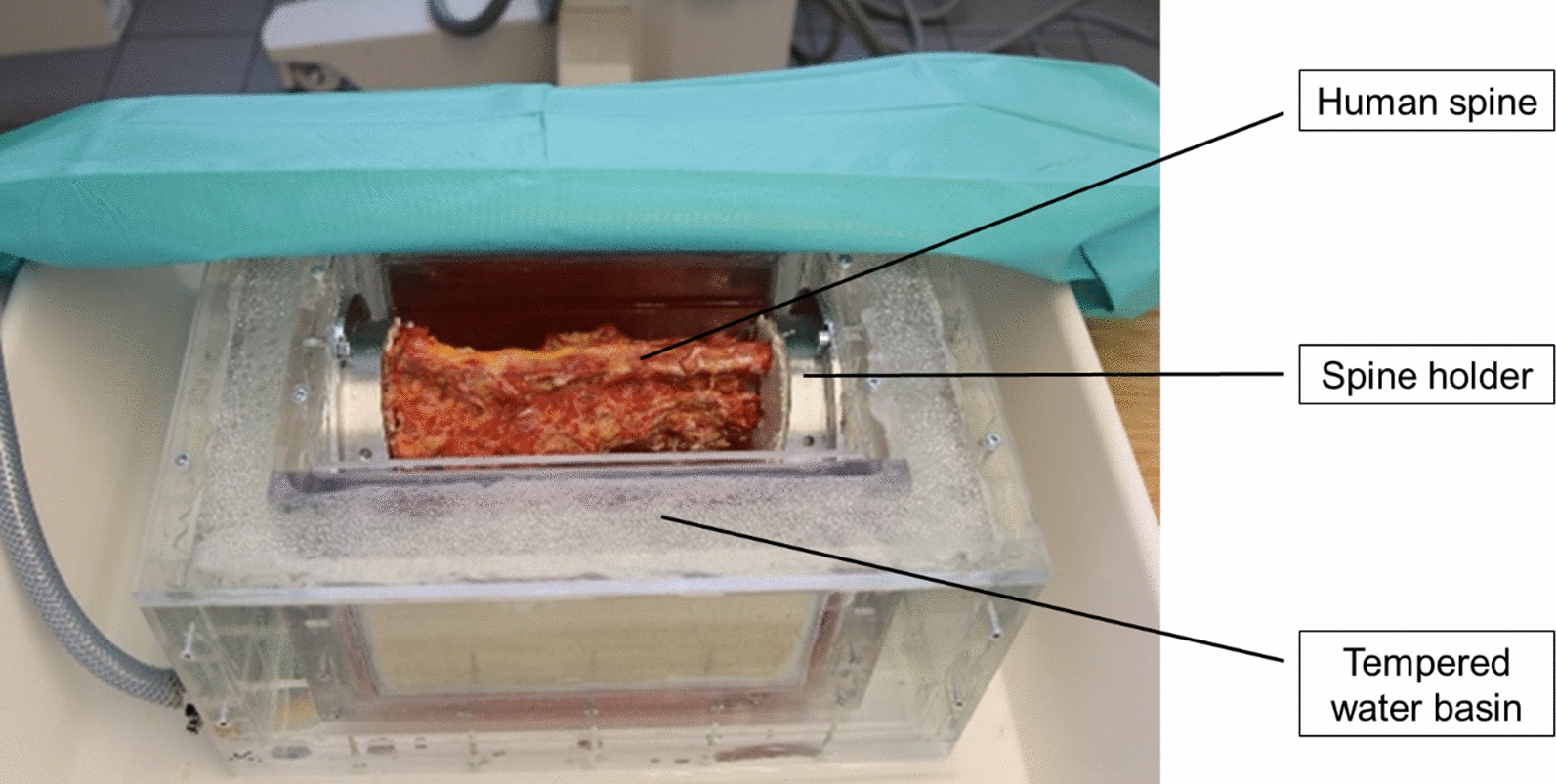
Test setup of the handling test

In a following evaluation, an experienced neurosurgeon and orthopedic surgeon assessed the handling of the functional samples and the anchoring mechanism on human specimens of the lumbar spine (Fig. 6). The focus was on implantation, setup mechanisms of the anchoring elements and imaging.

A questionnaire was developed to evaluate the handling, which had to be completed by the two experienced neurosurgeons. The following aspects were evaluated:

- Differences in handling and feeling during implantation and anchorage in the bone after implantation (before placement of the anchoring elements) compared to conventional standing screws.
- The anchorage situation after placement of the anchorage elements in comparison to cement augmentation.
- The additional work involved in setting up the anchoring mechanisms during implantation.
- The handling of the rotation mechanism for setting up the anchoring elements.
- The intraoperative imaging.

#### Test 3: Functional proof of the anchoring mechanism

**Fig. 7:**
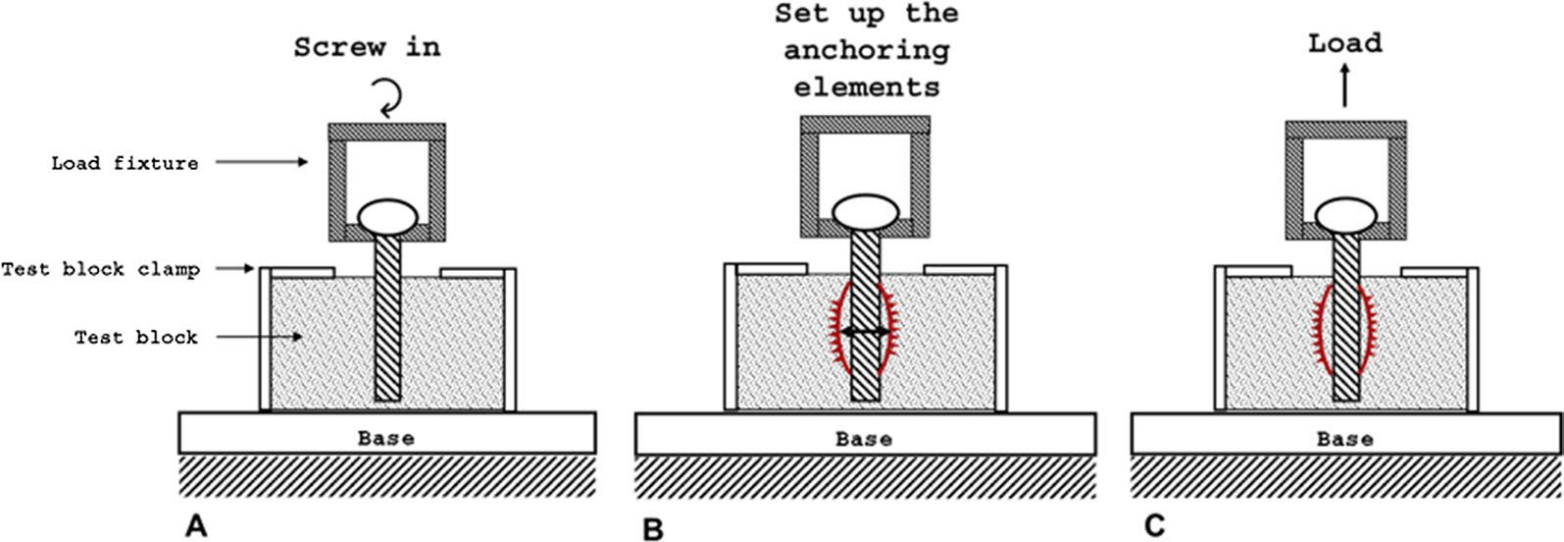
Pullout test setup. **A** Screwing in the screw; **B** setup the anchoring elements; *C* pullout the set up screw

A screw pullout test according to “ASTM F543_ Specification and Test methods for Metallic Medical Bone Screws” [33] in artificial bone (Block 10 PCF Cellular, SawBones, Sweden) was performed to investigate the increase in primary stability. The results of the NiTi functional samples were compared with those of the standard screw implants in terms of their pullout force and mechanical work. A 4-clamp jaw system was used to clamp the screws at the screw head. The artificial bone cube was fixed in a servo-electric static tension/compression and torsion testing machine (servo-hydraulic design 10 kN/200 Nm, DYNA-MESS Prüfsysteme GmbH, Germany) in a stainless steel test block clamp to prevent displacement. The screws were aligned with the center of the pre-drilled SawBone cube. At a speed of 18 /s and a feed rate of 2.5 mm/s, the screws were rotated 28 mm deep into the test specimen (Fig. 7A). After the screw was inserted into the artificial bone cube, the anchoring elements were set up so that the increase in screw diameter in the non-implanted state was 2 mm (Fig. 7B). This step was not necessary when testing the standard screws. The pullout speed was 0.083 mm/s. The screws were pulled out by 28 mm to ensure complete removal of the screw bodies (Fig. 7C).

For the pullout test, *N* = 12 standard and *N* = 12 NiTi screw implants were examined. For each screw implant, a separate artificial bone test block with a size of 400 × 400 × 800 mm was used. The artificial bone blocks were all from the same production batch (Block 10 PCF Cellular, SawBones, Sweden).

The evaluation of the results was carried out by means of a descriptive static analysis, evaluated using the median.

## Dicussion

For implantation and revision, conventional screwdrivers must be extended to mechanically set up the anchoring elements.

Furthermore, there is currently no locking mechanism installed for the maximum setup mechanisms of the anchoring elements, which prevents the elements from bending too much. This must take place in further adjustments to prevent the Ti6Al4V structures from flaking.

For further statements regarding the anchorage stability, toggle tests (ASTM 1717-15 [34]) must be carried out with human specimen, because pullout tests provide only limited information about the anchorage stability and optimal interlocking in the bone.

## Data Availability

The datasets supporting the conclusions of this article are included within the article.

## Abbreviations

NiTi: Nitinol
Fig.: Figure
